# Targeting AGTPBP1 inhibits pancreatic cancer progression via regulating microtubules and ERK signaling pathway

**DOI:** 10.1186/s10020-024-00892-x

**Published:** 2024-08-11

**Authors:** Ding-zhong Li, Zhe-yu Yang, Asi leng, Qian Zhang, Xiao-dong Zhang, Yan-chao Bian, Rui Xiao, Jian-jun Ren

**Affiliations:** 1 Department of Hepatobiliary, Pancreatic and Splenic Surgery, The Affiliated Hospital of Inner Mongolia Medical University, #1, Tongdao North Street, Huhhot, 010051 PR China; 2 Medical Simulation Center, Inner Mongolia Medical University, Huhhot, 010059 PR China; 3 Inner Mongolia Key Laboratory of Molecular Pathology, Inner Mongolia Medical University, #5, Xin Hua Street, Huhhot, 010059 PR China

**Keywords:** PDAC, Tubulin, AGTPBP1, MYLK, MAP1A

## Abstract

**Background:**

AGTPBP1 is a cytosolic carboxypeptidase that cleaves poly-glutamic acids from the C terminus or side chains of α/β tubulins. Although its dysregulated expression has been linked to the development of non-small cell lung cancer, the specific roles and mechanisms of AGTPBP1 in pancreatic cancer (PC) have yet to be fully understood. In this study, we examined the role of AGTPBP1 on PC in vitro and in vivo.

**Methods:**

Immunohistochemistry was used to examine the expression of AGTPBP1 in PC and non-cancerous tissues. Additionally, we assessed the malignant behaviors of PC cells following siRNA-mediated AGTPBP1 knockdown both in vitro and in vivo. RNA sequencing and bioinformatics analysis were performed to identify the differentially expressed genes regulated by AGTPBP1.

**Results:**

We determined that AGTPBP1 was overexpressed in PC tissues and the higher expression of AGTPBP1 was closely related to the location of tumors. AGTPBP1 inhibition can significantly decrease cell progression in vivo and in vitro. Moreover, the knockdown of AGTPBP1 inhibited the expression of ERK1/2, P-ERK1/2, MYLK, and TUBB4B proteins via the ERK signaling pathway.

**Conclusion:**

Our research indicates that AGTPBP1 may be a putative therapeutic target for PC.

**Supplementary Information:**

The online version contains supplementary material available at 10.1186/s10020-024-00892-x.

## Background

Pancreatic ductal adenocarcinoma (PDAC), the most common type of pancreatic cancer (PC), is a malignant tumor that originates from the ductal epithelium with a poor prognosis. A staggering over 85% of patients diagnosed with PC have already developed local or distant metastases, rendering surgical intervention impossible (Kleeff et al. 2016). According to a survey conducted on the incidence and mortality of 36 types of cancer in 185 countries around the world in 2020, PC was reported as the seventh leading cause of cancer death (Siegel et al. 2022).

Although radical surgery is still the primary treatment method for PC, patients are at a high risk of metastasis and have a 5-year survival rate of only 15-20% (Sung et al. 2021). Recent studies have shown that preoperative neoadjuvant chemotherapy can enhance the surgical resection rate in patients with locally advanced and borderline resectable PC (Rodríguez de la Vega Otazo et al. 2013; Von Hoff et al. 2013; Lopes and Maiato 2020), the overall effectiveness of this approach remains limited. Therefore, it is crucial to elucidate the underlying mechanism of PC in improving early detection rates for patients with PC and identify effective treatment strategies to enhance prognosis.

With the advancement of whole-genome sequencing technology, the molecular mechanisms underlying PC have become more accessible, and key driver genes such as KRAS, CDKN2A, TP53, and SMAD4/DPC4 have been identified (Hu et al. 2021). These genes are mutated in different stages of precursor lesions, and their dysregulation promotes the differentiation and proliferation of PCs. Despite numerous therapies targeting KRAS mutations, few can effectively suppress their effects and pathways in PC (Huang et al. 2021). Thus, it is paramountly important to identify new gene targets, clarify their roles, and develop effective therapeutic strategies for PDAC.

The ATP/GTP-binding protein 1 (AGTPBP1) gene, also known as the nervous system nuclear protein induced by axotomy (NNA1), encodes cytosolic carboxypeptidase 1 (CCP1) (Lalonde and Strazielle 2022). This gene has deglutamylase activity and functions to regulate the homeostasis of polyglutamylated tubulin in neurons by removing Glu residues at the C-terminus or side chains of α/β-tubulin (Berezniuk et al. 2012). AGTPBP1 mutations can lead to cerebellar ataxia due to Purkinje cell degeneration and the male mice displayed infertility (Mitchison and Kirschner 1984; Cassimeris et al. 1987; Nogales et al. 1999; Lin et al. 2020). In humans, there are six members of the cytosolic carboxypeptidases (CCPs) family including CCP1, CCP2, CCP3, CCP4, CCP5, and CCP6, similar to those in mice (Harris et al. 2000), all of which were reported to participate in deglutamylation of tubulin, which can regulate the assembly, transport and signal transduction of microtubules, and play an important role in maintaining the stability of microtubule cytoskeleton and function (Rodríguez de la Vega Otazo et al. 2013). Microtubules not only contribute to the formation of the cytoskeleton but also are crucial for controlling various essential cellular processes, such as cell division, shaping, motility, and intracellular transport (Janke and Magiera 2020).

AGTPBP1 has been thoroughly studied in neurodegenerative diseases (Kim et al. 2011; Shashi et al. 2018). However, the function of AGTPBP1 in cancers has not been focused on. The expression of AGTPBP1 in non-small cell lung cancer (NSCL) was correlated with cancer progression and immune invasion by bioinformatics analysis (Kwak et al. 2020), suggesting that this gene may be closely related to tumor progression.

In this study, we explored the expression and roles of AGTPBP1 in the progression of PDAC through in vivo and in vitro studies to uncover the underlying molecular mechanisms. Our findings will help to discover the new potential molecular markers of PDAC and provide the molecular targets for therapy.

## Methods

### Data sources and analysis

The cBioPortal for Cancer Genomics (cBioPortal, https://www.cbioportal.org/), a resource for interactive exploration of multidimensional cancer genomics data sets, was used to analyze the mutation status of AGTPBP1 across multiple cancer types. The Gene Expression Profiling Interactive Analysis tool (GEPIA, http://gepia.cancer-pku.cn/) and Human Protein Atlas database (https://www.proteinatlas.org/) were employed to examine the expression of AGTPBP1 in PDAC and normal tissues.

### Patient specimens collections

A total of forty cancerous and paracancerous normal tissues (a location over 2 cm away from the margin of the cancerous tissues) from PDAC patients who underwent surgery in the Affiliated Hospital of Inner Mongolia Medical University from 2018 to 2022 were collected. The sample inclusion criteria were: (1) Postoperative pathological examination confirmed as PDAC, (2) Patients did not receive radiotherapy, chemotherapy, immunotherapy, targeted therapy, and traditional Chinese medicine treatment before surgery, and (3) Patients have complete clinical data. The exclusion criteria were as follows: (1) PDAC combined with other malignancies; (2) The surgical tissue is too small to take, and (3) Postoperative pathological examination showed no PDAC. All tissue samples were obtained by the Ethics Committee of the Affiliated Hospital of Inner Mongolia Medical University [No. KY(2023014)], which follows the principles of the Declaration of Helsinki. All surgically resected specimens were stored in liquid nitrogen and fixed in formaldehyde for further study. Clinical data was collected for further statistical analysis.

#### Hematoxylin and eosin (HE) staining and immunohistochemical (IHC) staining

The tissue samples were fixed in 10% formalin, dehydrated with different graded alcohol series, embedded in paraffin, and cut into 4 μm sections. The sections were stained with hematoxylin staining for 2 min and eosin for 1 min. Finally, the slides were scanned by the Aperio LV1 digital pathological scanner (Leica, Germany), and the images were analyzed by Image-Pro Plus software (Bioimager Inc., Canada).

IHC was performed using the Strept Avidin Biotin-peroxidase Complex (SABC) method according to the manufacturer’s instructions (SABC immunohistochemical staining kit, Wuhan Bode Bioengineering Co. Ltd., China). Briefly, after deparaffinization with xylene and dehydration with an alcohol gradient, 3% H_2_O_2_ was applied for 10 min to suppress endogenous peroxidase activity. Subsequently, the antigen retrieval protocol was performed for 20 min at 98 °C. After incubation with 5% BSA for 30 min at 37 °C to block non-specific binding, the slides were incubated with the primary antibody AGTPBP1 (1:200, 14067-1-AP, Proteintech Ltd., USA), Ki-67 (ready-to-use antibody, RMA-0731, Maixin Biotechnology Development Co., Ltd., China), and CD31 (1:2000, EPR17259, Abcam Corporation, USA) overnight at 4 °C, followed by a biotin-conjugated secondary antibody for 30 min at 37 °C. After incubation with SABC reagent, the slides were proceeded with the DAB kit. Finally, the mounted slides were observed under a microscope. The average optical density (AOD) of stained tissues was measured from five different view fields using Image Pro Plus software to determine protein expression levels.

### RNA extraction and reverse transcription quantitative PCR (RT-qPCR)

The total RNA was extracted using the Trizol reagent following the manufacturer’s protocol (Tiangen Biotech, China). The RNA concentration was measured for the yield and purity in NanoDrop^™^ 2000 C (Thermo Fisher Scientific, USA). Reverse transcription was carried out using TB Green Premix Ex Taq II (Tli RNaseH Plus) (Takara, Japan) with 500 ng total RNA, then 2 µL cDNA was used for the RT-qPCR amplification using Applied Biosystems^™^ 7500fast Real-Time PCR System (Thermo Fisher Scientific, USA). qPCR conditions were used as follows: 40 cycles of denaturation at 95 °C for 5 s, annealing at 60 °C for 34 s, and dissociation stage. The Relative expression was calculated using the 2^−ΔΔCt^ method and the reference gene glyceraldehyde-3-phosphate dehydrogenase (GAPDH) was used as an internal control.

### Protein extraction and western blot

The protein was isolated using radioimmunoprecipitation assay (RIPA) lysis buffer with 100X PMSF (Beyotime Biotechnology, China) and the concentration was measured using a bicinchoninic acid assay (BCA) kit (Beyotime Biotechnology, China). A 20 µg protein lysate was separated by 10% SDS-PAGE and electrotransferred to a nitrocellulose (NC) membrane (Millipore, USA). The membranes were blocked with 5% skim milk for 3 h at room temperature. After that, they were incubated at 4 °C overnight with the specified primary antibodies as follows: AGTPBP1 (1:1000), GAPDH (1:5000), ERK1/2(1:1000), phospho-ERK1/2 (P-ERK1/2, 1:1000) and TUBB4B (1:1000) from ProteinTech, USA. The next day, the membranes were washed with TBST (3 × 10 min) and incubated with the IRDye^®^ 800CW Goat anti-Rabbit or anti-Mouse IgG secondary antibody (1:10000) for 1 h at room temperature. After washing, the protein bands were then detected and visualized by Odyssey^®^ DLx Imaging System (LI-COR, USA), and the image intensity was analyzed with the ImageJ software (https://imagej.net).

#### Cell culture and small interfering RNAs (siRNAs) transfection

Human pancreatic ductal epithelial cells HPDE6-C7 and pancreatic cancer cell lines PANC-1 and HPAF-II were purchased from the BeNa Culture Collection (Beijing Beinachuang Biotechnology Research Institute, China). The cells were cultured in DMEM high glucose medium supplemented with 10% fetal bovine serum (FBS) and 1% penicillin-streptomycin at 37°C with 5% CO_2_. The three FAM-labeled siRNAs for AGTPBP1 and scrambled negative control (NC) were synthesized by Sangon Co. Ltd. (Shanghai, China) and transfected into PANC-1 cells using Lipofectamine^™^ 2000 (Thermo Fisher Scientific, USA) by the manufacturer’s instructions. The siRNA sequences targeting AGTPBP1 were as follows: siAGTPBP1-1050: sense 5’-FAM-AGAUUGGCACCGCCAUGAUAATT-3’ and antisense 5’-FAM-UUAUCAUGGCGGUGCCAAUCUTT-3’; siAGTPBP1-2136: sense 5’-FAM-GCCUCCAUUCAAAGAGCCUAUTT-3’ and antisense 5’-FAM-AUAGGCUCUUUGAAUGGAGGCTT-3’; siAGTPBP1-3463: sense 5’-FAM-UAUACCAUGGAGAGUACUUUATT-3’ and antisense 5’-FAM-UAAAGUACUCUCCAUGGUAUATT-3’; NC: sense 5’-FAM-UUCUCCGAACGUGUCACGUTT-3’ and antisense 5’-FAM-ACGUGACACGUUCGGAGAATT-3’. The siAGTPBP1-1050 (referred to as sh-AGTPBP1) with a better silencing effect compared to other siRNAs was selected for further study.

#### Cell counting Kit-8 assay (CCK-8 )

CCK-8 kit was used to detect the proliferation of PANC-1 cells transfected with siAGTPBP1-1050 following the manufacturer’s instructions (Vazyme Biotech, China). Briefly, the cells were seeded in a 96-well plate with 3 × 10^4^ cells/well. 10 µl of CCK-8 reagent was added to each well at 24 h, 48 h, and 72 h, and then incubated at 37 °C for 2 h. Finally, the absorbance of cells at 450 nm was measured by a microplate reader (Biotek ELx800, USA). All assays were independently repeated 5 times (*N* = 5).

#### Plate colony formation assays

The cells were transfected with siAGTPBP1-1050 and NC, respectively. Then the cells were digested and seeded in 90 mm dishes with 2 × 10^3^ cells/dish and continued to incubate until the colonies of cells in the dish can be observed. After the medium was discarded, cells were washed with PBS, and fixed with 4% paraformaldehyde, After stained with 0.1% crystal violet for 10 min at room temperature, the colonies were washed again with PBS and air-dried. Finally, the colonies were photographed and analyzed. All assays were independently repeated 3 times (*N* = 3).

### Wound healing assays

Wound healing assays were performed to measure the migration ability of PANC-1 cells after AGTPBP1 knockdown. The transfected cells (siRNA-AGTPBP1) were grown in the 6-well plates to reach over 90% confluency, and a scratch was made with a pipette tip to create an incision-like gap. Floating dead cells were washed away with PBS and replaced with a serum-free basal medium. The “wounded” area was photographed immediately after wounding and at 24 h thereafter, and cell migration was quantified and expressed as the average percentage of closure of the scratch area by ImageJ software. All assays were independently repeated 3 times (*N* = 3).

### Transwell migration and invasion assay

Transfected PANC-1 cells were analyzed using Transwell chambers (Corning, USA) with Matrigel (BD, USA). A 200ul of 2.5 × 10^5^ cells/mL with serum-free basal medium was added to the upper chamber. The same cell number was used in the siRNA-AGTPBP1 and NC groups. The lower chamber was filled with a complete medium containing 10% FBS as a chemoattractant. Matrigel was not added in the cell migration experiment but added in the cell invasion experiment. The chambers were removed after 24 h of incubation and processed for 4% paraformaldehyde fixation and 0.1% crystal violet staining for 30 min. The migrated and invaded cells were counted and imaged by an inverted microscopy (IX73, Olympus, Japan) after air-drying. All assays were independently repeated 3 times (*N* = 3).

### Cell cycle analysis by flow cytometry

The transfected cells were digested with EDTA-free trypsin, resuspended in the pre-chilled PBS, and fixed in 70% ethanol at 4 °C overnight. On the following day, they were centrifuged and washed with pre-chilled PBS. After centrifugation and removal of PBS, propidium iodide (PI) was added to the transfected cells and incubated at 37 °C in the dark for 30 min. The cell cycle was assessed using the NovoCyte flow cytometer (Agilent Technologies, USA) according to the manufacturer’s instructions. All experiments were independently repeated 3 times.

### RNA sequencing (RNA-seq) and data analysis

Total RNA was extracted from two groups of cells (siRNA-AGTPBP1 and negative control NC, *n* = 3) using TRIzol^®^ Reagent according to the manufacturer’s instructions (Magen). RNA samples were detected based on the A260/A280 absorbance ratio with a Nanodrop ND-2000 system (Thermo Scientific, USA), and the RNA integrity number (RIN) of RNA was determined by an Agilent Bioanalyzer 4150 system (Agilent Technologies, CA, USA). Paired-end libraries were prepared using an ABclonal mRNA-seq Lib Prep Kit (ABclonal, China) following the manufacturer’s instructions. Adaptor-ligated cDNA was used for PCR amplification. PCR products were purified (AMPure XP system) and library quality was assessed on an Agilent Bioanalyzer 4150 system. Finally, the library preparations were sequenced on an MGISEQ-T7 (BGI, China) by Applied Protein Biotechnology (Shanghai, China), and 150 bp paired-end reads were generated. The data generated from the BGI platform were used for bioinformatics analysis. All of the analyses were performed using an in-house pipeline from Shanghai Applied Protein Technology.

Raw data of fastq format were firstly processed through in-house Perl scripts. In this step, remove the adapter sequence and filter out low quality (low quality, the number of lines with a string quality value less than or equal to 25 accounts for more than 60% of the entire reading) and N (N means that the base information cannot be determined) ratio is greater than 5% reads to obtain clean reads that can be used for subsequent analysis. Then clean reads were separately aligned to the reference genome with orientation mode using HISAT2 software (http://daehwankimlab.github.io/hisat2/) to obtain mapped reads. FeatureCounts (http://subread.sourceforge.net/) was used to count the reads numbers mapped to each gene. Then FPKM of each gene was calculated based on the length of the gene and the reads count mapped to this gene.

Differential expression analysis was performed using the DESeq2 (http://bioconductor.org/packages/release/bioc/html/DESeq2.html), the differentially expressed genes (DEGs) with |log2FC| > 1 and Padj < 0.05 were considered to be significantly different.

The Gene Ontology (GO) and Kyoto Encyclopedia of Genes and Genomes (KEGG) enrichment analysis of DEGs were performed to identify the significant pathways at the gene function level. The clusterProfiler R software package for GO function and KEGG pathway enrichment analysis was used. *P* < 0.05 was considered significantly enriched.

### In vivo tumor model

The five-week-old *BALB/c* male nude mice were purchased from the Vital River, Beijing, China. Nude mice were randomly divided into two groups (siAGTPBP1 group and NC group, *n* = 6). A total of 100 cell suspension with a concentration of 1 × 10^8^ cells/mL was injected subcutaneously into the axilla of nude mice. The tumor formation was observed at 12 weeks continually. Then the nude mice were euthanized and the subcutaneous tumor tissue was carefully collected. The length and width of tumor tissue were measured with calipers, and the volume of the tumor was calculated according to the formula: V=[(length×width×2)/2.

### Statistical analysis

Data were statistically analyzed and graphed using SPSS 25.0 and GraphPad Prism 8.0 (La Jolla, CA, USA). The χ2 test was used to analyze the correlation of AGTPBP1 expression with clinical pathological parameters. For the two sets of measurement data, when the normal distribution is consistent and the variance is homogeneous, the Student’s t-test is used; If the normal distribution is not satisfied, a nonparametric test is used. All data are measured as mean ± standard deviation (SD). *P* < 0.05 was statistically significant.

## Results

### AGTPBP1 is up-regulated in PC

Tumorigenesis is often caused by gene mutations such as KRAS and p53 (Shan et al. 2020). These mutations are enriched in cancers. However, their functional importance remains hard to predict. Thus, in our study, to explore AGTPBP1 function in PC, we firstly examined its mutations, structural variant, and putative copy-number alterations (CNAs) from the GISTIC (Genomic Identification of Significant Targets in Cancer) algorithm across cancers in 5 pancreatic adenocarcinoma studies with 988 samples using the cBioPortal analysis. As a result, two missense (M205L and L552F) and one nonsense (G98*) mutations of AGTPBP1 were identified from 2 out of 5 studies (Supplementary Fig. 1A). Although the mutation frequency is very low (< 1%), these mutations occurred in AGTPBP1 may be crucial for PC development, however, it needs to be proved experimentally in further study.

Subsequently, we examined the expression of AGTPBP1 in 179 pancreatic cancer and 171 pancreatic normal tissues by GEPIA, an online gene expression interaction analysis database based on the TCGA database and GTEx project. The analysis results showed that the expression of AGTPBP1 in PC tissues was up-regulated compared with that in normal pancreatic tissues (Supplementary Fig. 1B) (http://gepia.cancer-pku.cn/). The Human Protein Atlas database (https://www.proteinatlas.org/) was used to analyze the protein expression of AGTPBP1 in PC tissues and normal pancreatic tissues. The analysis showed that AGTPBP1 in PC tissues were significantly higher than that in paracancerous tissues (Supplementary Fig. 1C). Furthermore, the study revealed a high expression of AGTPBP1 in endothelial cells and ductal cells of pancreatic tissues (Supplementary Fig. 1D).

**Fig. 1:**
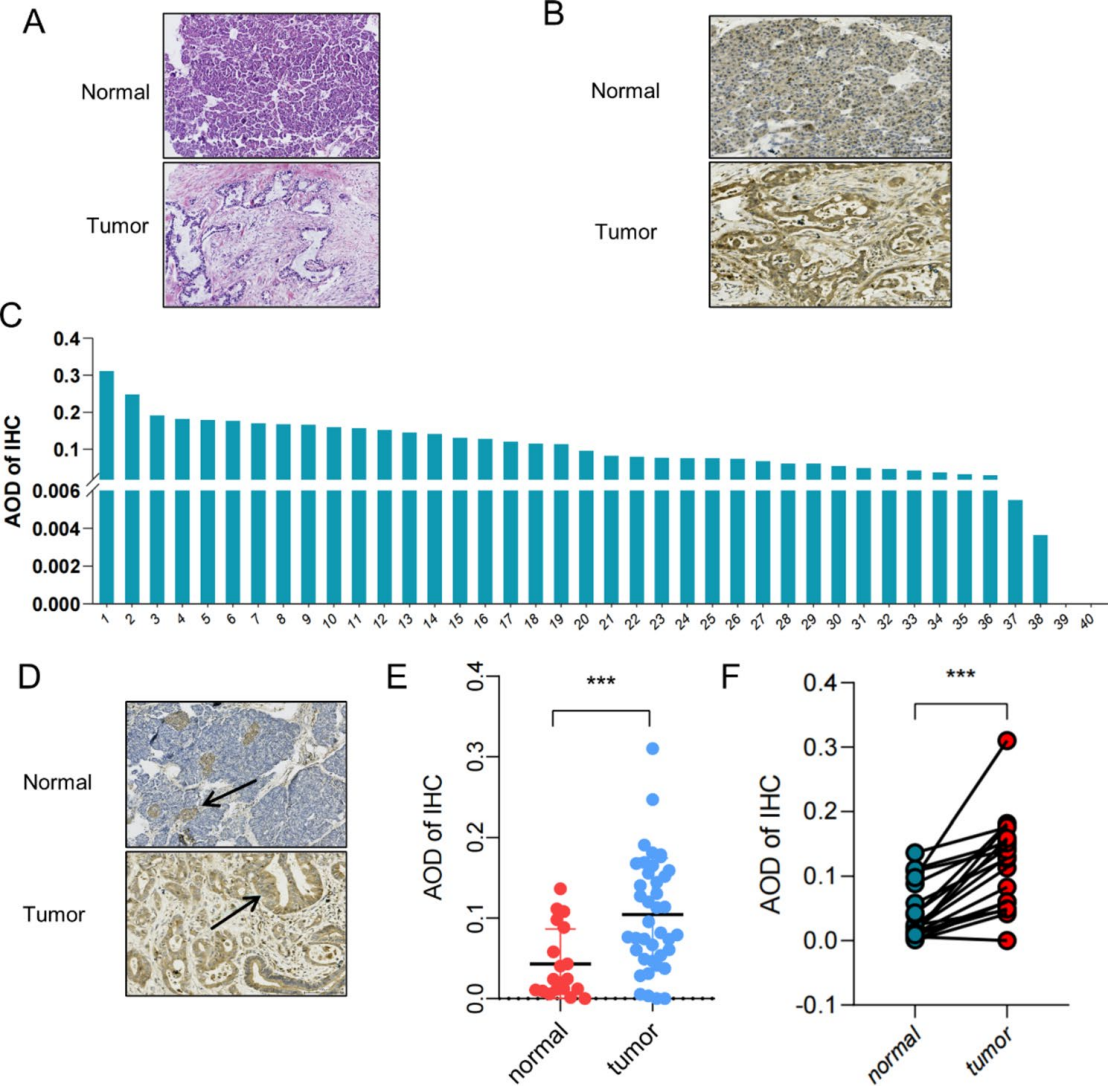
Expression of AGTPBP1 in PDAC tissues. **A** HE staining of cancer tissue and precancerous normal tissues of PDAC patients was conducted. Scale bar, 100 μm. **B** Immunohistochemical staining using anti-AGTPBP1 antibody showed higher expression in PDAC than normal pancreatic tissues. Scale bar, 100 μm. **C** The average optical density (AOD) of AGTPBP1 immunohistochemical staining in the cancer tissues of 40 PDAC patients was measured. **D** AGTPBP1 immunohistochemical staining showed that it was mainly expressed in pancreatic islets in normal pancreatic tissues, while it was mainly expressed in pancreatic ductal epithelial cells in PDAC tissues. Scale bar, 100 μm. **E** Expression of AGTPBP1 in 40 PDAC and 19 normal pancreatic tissues. **F** Expression of AGTPBP1 in 19 pairs of PDAC and normal pancreatic tissues. All data are presented as means ± SD. The Student’s t-test was used to compare the means between the two groups. ***P < 0.001 versus (vs.) normal group

To further confirm the expression of AGTPBP1 in PDAC and adjacent normal pancreatic tissues, we collected 40 PDAC and 19 normal pancreatic tissue samples from surgical resections and subsequently performed HE (Fig. 1A) and IHC staining assays (Fig. 1B-F). IHC analysis based on the AOD measurements revealed AGTPBP1 was highly expressed in PDAC compared to normal tissues with significant heterogeneity of AGTPBP1 expression in both PDAC and pancreatic normal tissues (Fig. 1B, C, and E). In normal pancreatic tissues, AGTPBP1 expression was predominantly localized to the islets, as depicted in Fig. 1D (arrow indicated). In contrast, within pancreatic ductal adenocarcinoma cells, AGTPBP1 was primarily observed in the cytoplasm (Fig. 1D, arrow indicated), with only rare occurrences of nuclear localization. Out of the 40, 19 samples were paired and then we compared the expression of the AGTPBP1 gene in these 19 pairs (Fig. 1F). The results showed that 18 of the paired PDAC tissues had higher expression of the AGTPBP1 gene (*P* < 0.001), while only 1 pair showed the opposite result. Our analysis demonstrated a significant increase in AGTPBP1 expression in cancerous tissues of PDAC patients compared to normal pancreatic tissue, indicating the crucial role of AGTPBP1 in PDAC.

### Up-regulation of AGTPBP1 in PDAC is correlated with the location of the tumor

To further explore the clinical significance of AGTPBP1 overexpression in PC, clinicopathological characteristics of 40 PC specimens are summarized in Table 1. Chi-square ($X^2$) test analysis results showed that the expression of AGTPBP1 was significantly correlated with the location of the tumor ($\:P=0.028$), but no notable correlation between AGTPBP1 expression and patient’s gender, age, clinicopathologic grades, extent of resection, or tumor size in 40 cancerous cases. The higher expression of AGTPBP1 correlated with tumor located at the tail of the pancreatic body and lower expression at the pancreatic head and hook (Table 1).

**Table 1:** Relationship between the expression level of AGTPBP1 and clinical features in 40 patients with pancreatic cancer

		AGTPBP1 expression		
Parameters	NO.(%)	High^a^ (*n* = 19)	Low^a^ (*n* = 21)	P Value
Gender				0.356
Male	22(55)	9(22.5)	13(32.5)	
Female	18(45)	10(25)	8(20)	
Age(Years)				0.873
<60	11(27.5)	5(12.5)	6(15)	
≥ 60	29(72.5)	14(35)	15(37.5)	
Differentiation				0.070
Poor	19(47.5)	6(15)	13(32.5)	
Moderate	9(22.5)	4(10)	5(12.5)	
Well	12(30)	9(22.5)	3(7.5)	
TNM Stage^b^				0.664
I、II	34(85)	17(42.5)	17(42.5)	
III、IV	6(15)	2(5)	4(10)	
Neural Invasion				0.148
Yes	30(75)	12(30)	18(45)	
No	10(25)	7(17.5)	3(7.5)	
Lymph Node Metastasis				0.987
Yes	21(52.5)	10(25)	11(27.5)	
No	19(47.5)	9(22.5)	10(25)	
CEA				0.119
<5ug/L	22(55)	8(20)	14(35)	
≥ 5ug/L	18(45)	11(27.5)	7(17.5)	
CA199				0.442
<37U/ml	8(20)	5(12.5)	3(7.5)	
≥ 37U/ml	32(80)	14(35)	18(45)	
Tumor Location				0.028^c^
Head and neck	24(60)	8(20)	16(40)	
Body and tail	16(40)	11(27.5)	5(12.5)	
History of Diabetes				0.172
Yes	5(12.5)	4(10)	1(2.5)	
No	35(87.5)	15(37.5)	20(50)	

a Forty patients with pancreatic cancer were divided into a high-expression group and a low group according to the mean expression of AGTPBP1 in pancreatic cancer tissue

b According to the 8th Edition of the AJCC Cancer Manual

c Statistically significant, *P* < 0.05

### Knockdown of AGTPBP1 can significantly inhibit the malignant biological behaviors of pancreatic cancer cells

**Fig. 2:**
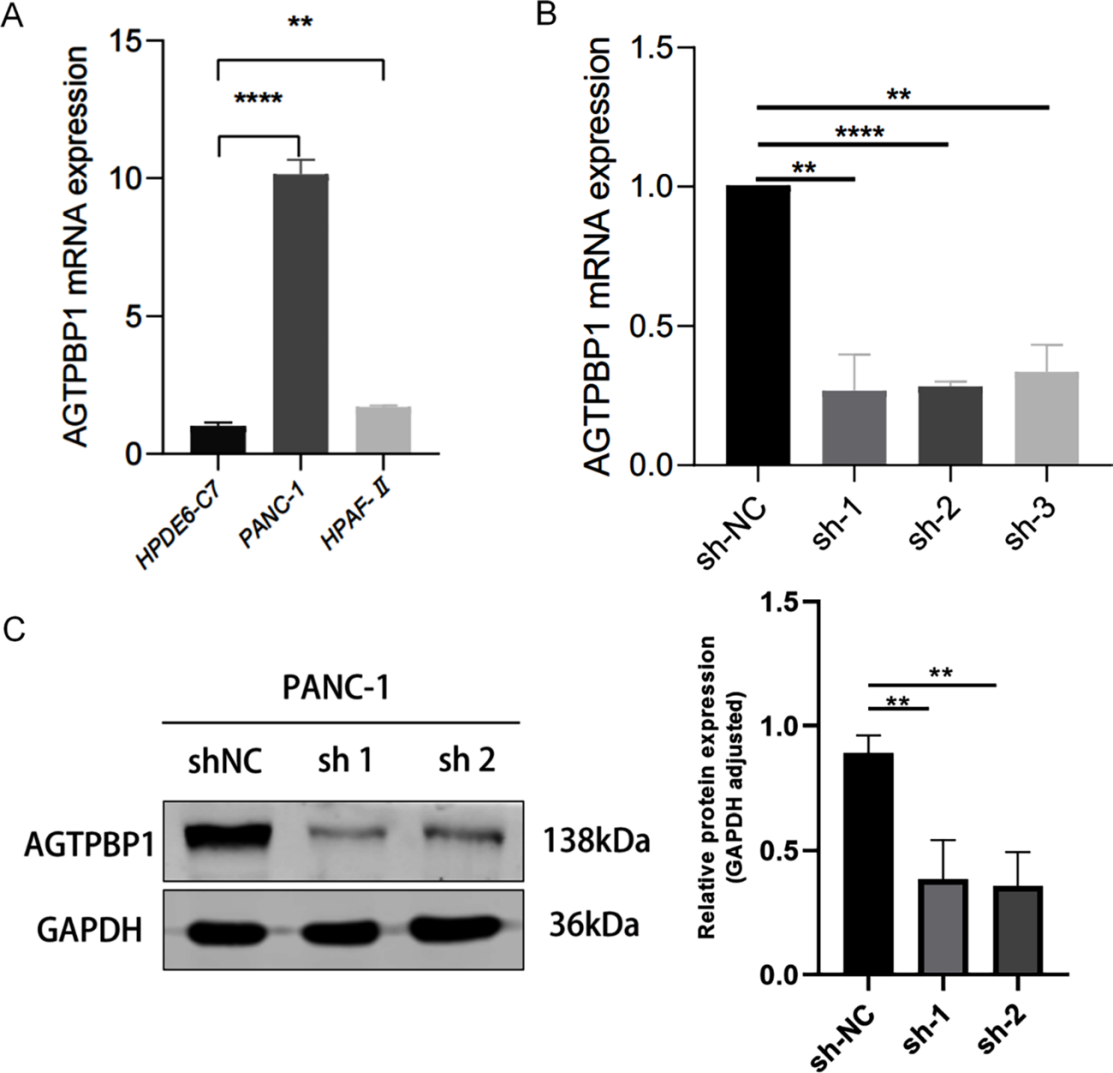
**Expression of AGTPBP1 in human pancreatic cancer cell lines and siRNA inhibition of AGTPBP1 expression in PANC-1 cells. A** The expression of AGTPBP1 was up-regulated in PANC-1 and HPAF-II PC cells compared with normal control pancreatic endothelial cells by RT-qPCR. **P < 0.01, ****P < 0.0001 vs. HPDE6-C7. **B-C** The siRNA-1(targeting AGTPBP1-1050) and siRNA-2 (targeting AGTPBP1-2136) could inhibit AGTPBP1 expression. **B** The relative expression of AGTPBP1 by RT-qPCR. **C** Western blot analysis of AGTPBP1 expression. All data are presented as means ± SD. The Student’s t-test was used for statistical comparison. All experiments were performed in triplicate. **P < 0.0001, ****P < 0.01 vs. sh-NC

Next, we investigated the expression of AGTPBP1 in human normal pancreatic ductal epithelial cells (HPDE6-C7) and pancreatic cancer cells such as PANC-1 and HPAF-II using RT-qPCR. Our findings revealed that the expression of AGTPBP1 was significantly higher in pancreatic cancer cells (PANC-1 and HPAF-II) compared to HPDE6-C7 cells. Furthermore, the expression of AGTPBP1 was higher in PANC-1 than in HPAF-II cells (Fig. 2A). Thus, we chose to use PANC-1 cells for further functional analysis.

PANC-1 cells were transfected with three siRNA sequences targeting AGTPBP1 and control siRNA (sh-NC) for 48 h to evaluate the efficiency of transfection. Immunofluorescence observation, RT-qPCR, and western blot analysis were used to determine the knockdown efficiency of each siRNA. As a result, siRNA-1050 (sh-1) and siRNA-2136 (sh-2) effectively reduced AGTPBP1 expression compared to siRNA-3463 (sh-3) in the PANC-1 cells (Fig. 2B). We conducted a western blot to evaluate the effect of AGTPBP1 knockdown and observed that siRNA-1050 (sh-1) had a stronger inhibition effect than siRNA-2136 (sh-2) (Fig. 2C). Based on this result, we decided to use the PANC-1 cells and siRNA-1050 (sh-1) to investigate the effect of AGTPBP1 knockout on PC malignant biological behaviors.

**Fig. 3:**
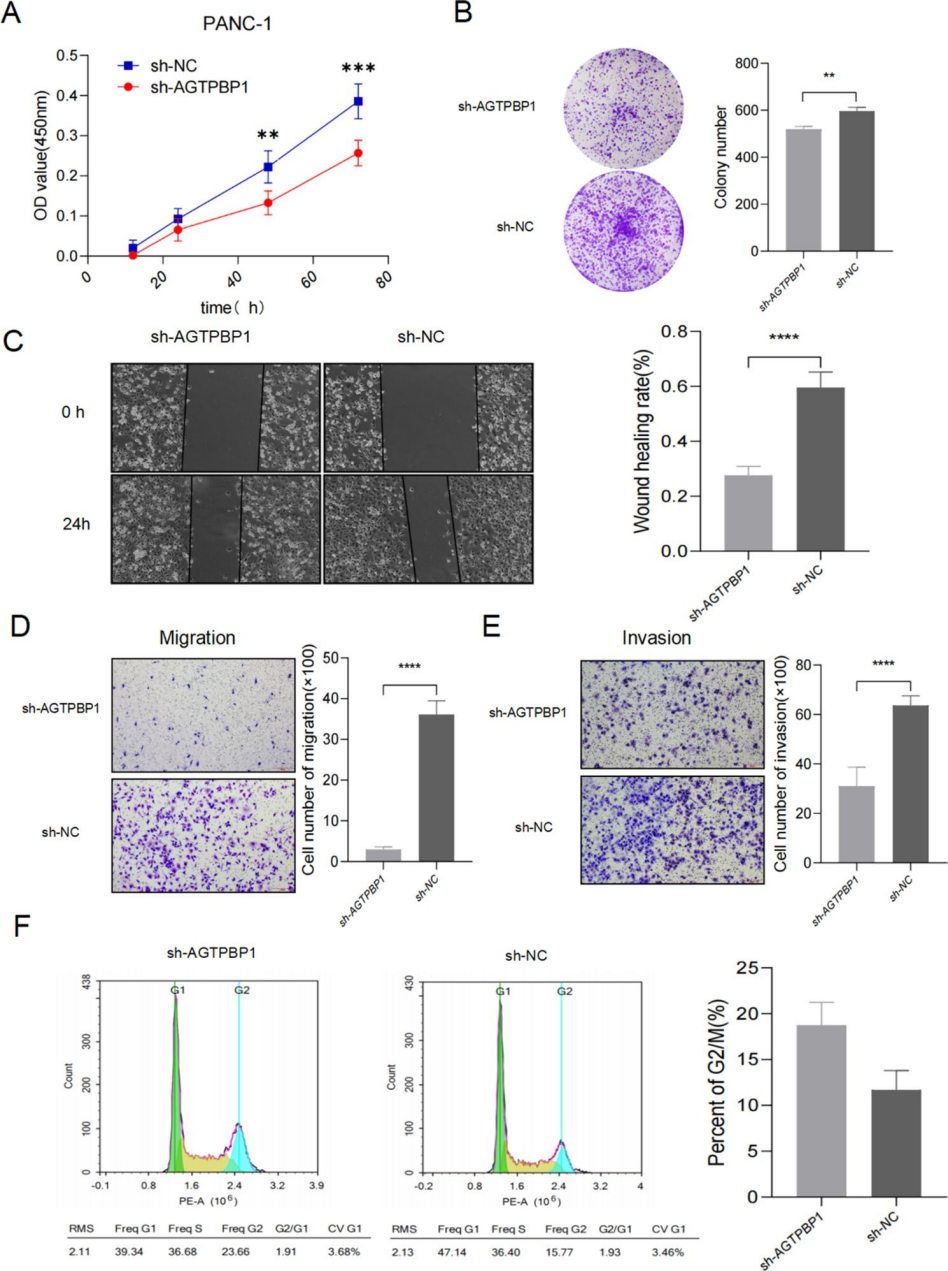
**Knockdown of AGTPBP1 inhibits the malignant biological behaviors of pancreatic cancer cells in vitro A** The proliferation experiments using CCK-8 cells showed that knocking out AGTPBP1 in PANC-1 cells could inhibit its proliferation ability (*n* = 5). **B** Plate clonalization experiments showed that knocking out AGTPBP1 significantly inhibited the formation of colonies of pancreatic cancer cells, counting the number of colonies containing more than 50 cells (*n* = 3). Scale bar, 200 μm. **C** Cell scratch healing experiments showed that knocking out AGTPBP1 significantly inhibited the migration ability of pancreatic cancer cells (*n* = 3). Scale bar, 200 μm. Transwell compartment migration and invasion experiments showed that knocking down AGTPBP1 could significantly reduce the migration (**D**) and invasion (**E**) ability of pancreatic cancer cells (*n* = 3). Scale bar, 200 μm. **F** After knocking out AGTPBP1 for pancreatic cancer cells, cells are blocked in the G2/M phase (*n* = 3). All results are representative of three independent experiments. Values are presented as mean ± SD. The Student’s t-test was used to compare the means between the two groups. **P<0.01,***P<0.001,****P<0.0001 vs. sh-NC

We carried out the CCK-8 and plate colony formation assays to evaluate the effect of AGTPBP1 interference on the proliferation of PANC-1 cells. As shown in Fig. 3A and B, inhibition of AGTPBP1 (sh-AGTPBP1) remarkably reduced the proliferation of PANC-1 cells compared to the cells transfected with control siRNA (sh-NC) ($\:P<0.01$). The plate colony formation assays confirmed the results obtained by CCK-8 assays ($\:P<0.01$) (Fig. 3B). It meant that slicing AGTPBP1 expression did affect cell proliferation. Whether reduced AGTPBP1 could inhibit cell migration and invasion, we analyzed the potential function of AGTPBP1 to induce cell motility via wound healing and trans-well assays. In the wound-healing assay, we observed fewer cells migrating into the wound area in sh-AGTPBP1 transfected PANC-1 cells than in control cells treated with sh-NC (Fig. 3C) ($\:P<0.0001$), which indicated that the migration ability of AGTPBP1 knockdown (KD) PANC-1 cells was decreased compared to the negative control cells (Fig. 3C). The transwell assays confirmed that AGTPBP1 interference could inhibit the migration ability of PANC-1 (Fig. 3D) ($\:P<0.0001$). Meanwhile, the results in the transwell migration assay also revealed that the number of PANC-1 cells that passed through the membrane onto the lower chamber was dramatically inhibited in the sh-AGTPBP1 transfected cells compared to the control (Fig. 3E) ($\:P<0.0001$), indicating that knockdown of AGTPBP1 significantly inhibited the invasion ability of PANC-1 cells. These findings underscore that AGTPBP1 did play an important role in PC cell proliferation, migration, and invasion.

In addition, to further explore the mechanism of AGTPBP1 on the proliferation of PC cells, the flow cytometric assay of the cell cycle distribution was investigated and exhibited that AGTPBP1 knockdown dramatically decreased the percentage of the G1 phase from 47.14 to 39.34% and increased the percentage of G2 phase cells from 15.77 to 23.66%, respectively. The PC cells after AGTPBP1 knockdown were arrested at the G2/M phase compared with the control cells (*P*<0.01) (Fig. 3F). The cell cycle analysis demonstrated that the silencing of AGTPBP1 caused the G2/M phase arrest of PANC-1, which implies that the silencing of AGTPBP1 in PANC-1 blocked the transition of the G2/M phase of the cell cycle. Altogether, these results suggest that AGTPBP1 might play an important role in the growth and survival of PC cells. In summary, our study revealed that decreased expression of AGTPBP1 can significantly inhibit the malignant biological behaviors of PANC-1 cells.

**Knockdown of AGTPBP1 can attenuate the xenograft tumor growth and new blood vessel formation in nude mice**.

**Fig. 4:**
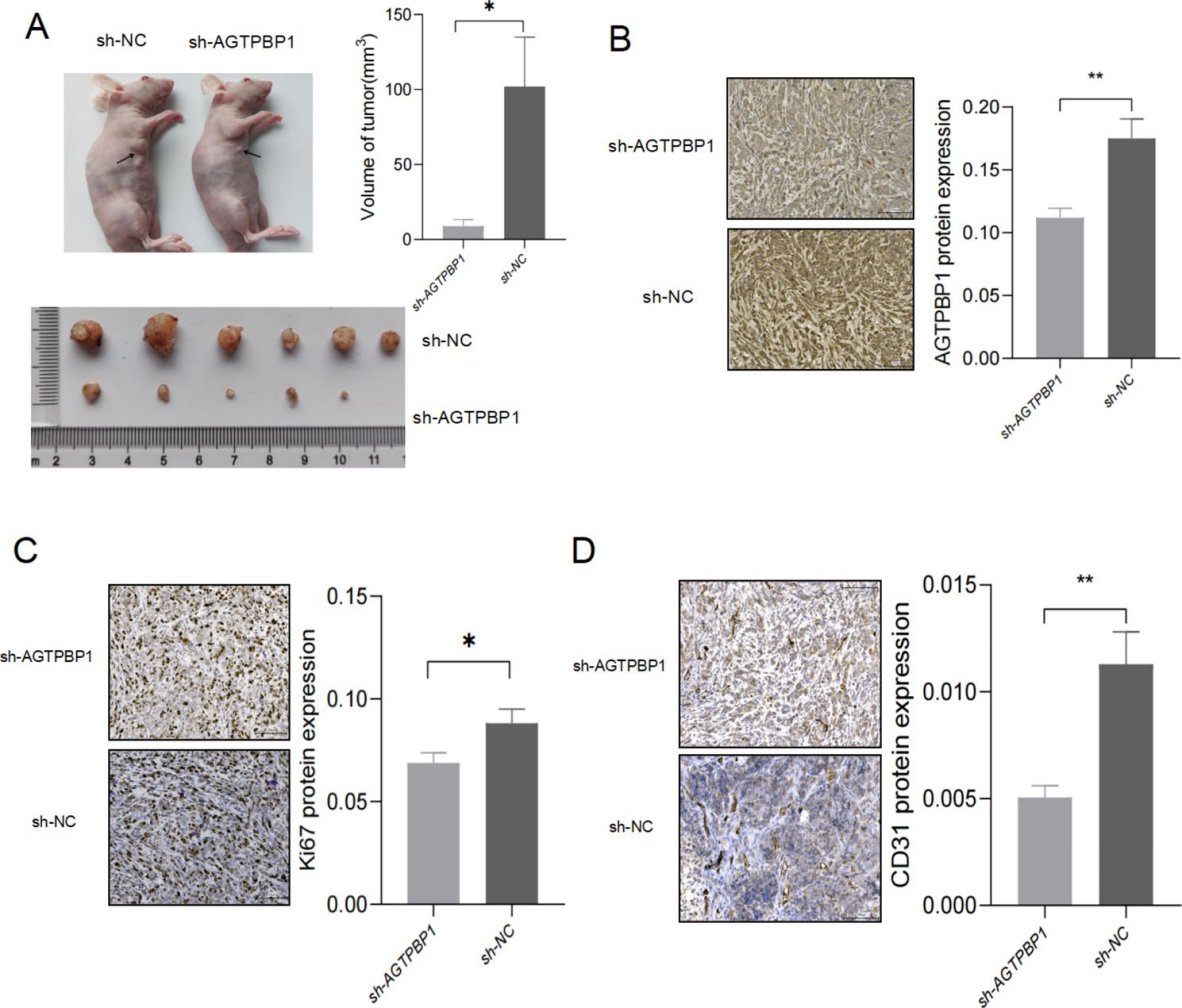
**Knockdown of AGTPBP1 can inhibit the growth of xenograft tumors and the formation of new blood vessels in pancreatic cancer cells**. **A** Comparative tumor manifestations and volume of subcutaneous tumorigenesis in the PANC-1 cell line AGTPBP1 KD group (*n* = 5) and the control group (*n* = 6). **B-D** Representative images from IHC staining of AGTPBP1 (B), Ki67 (C), and CD31 (D) expression in xenograft tumors. The positive signal was measured by ImageJ. Scale bar, 100 μm. Values are presented as mean ± SD. The Student’s t-test was used to compare the means between the two groups. *(*P* < 0.05), **P<0.01 vs. sh-NC

To assess the ability of AGTPBP1 knockdown to inhibit the tumorigenic capacity of PC, sh-AGTPBP1 and sh-NC PANC-1 cells were subcutaneously injected into 5-week-old *Balb/c* nude mice (1.5 × 10^6^ cells/200 µl PBS/animal). The xenograft tumors were formed 60 days after injection, the nude mice were euthanized after 30 days of continuous observation and the tumors were completely dissected. There was a significant decrease in the sh-AGTPBP1 group in tumor size compared with their control sh-NC counterparts (*P* < 0.05) (Fig. 4A). HE staining of the tumor samples showed that blood vessel was formed in the control mice injected with sh-NC cells. However, much fewer blood vessels existed in the AGTPBP1 KD tumors, which indicates that AGTPBP1 was required for in vivo differentiation of PANC-1 to form blood vessels. Thus, we performed the IHC for xenograft tumors using AGTPBP1, Ki67, and CD31 antibodies to evaluate the tumorigenesis in vivo. Human CD31 antibody was used to detect blood vessels and Ki-67 was used as the proliferation marker for tumors. As shown in Fig. 4B, the expression of AGTPBP1 was significantly down-regulated in the xenografted tumors formed by injection of sh-AGTPBP1 cells than that in the control counterparts (Fig. 4B) (*P* < 0.01). The expression level of Ki67 in the sh-AGTPBP1 tumors was also decreased than that in the control group, consistent with the results obtained in vitro (Fig. 4C) (*P* < 0.05). CD31 was mainly expressed in neovascular endothelial cells and the expression level of CD31 in tumors with sh-AGTPBP1 was significantly lower than that in the control group, indicating that knockdown of AGTPBP1 in PANC-1 could significantly reduce the formation of new blood vessels in xenograft tumors (Fig. 4D) (*P* < 0.01). Our results confirmed that AGTPBP1 knockdown could attenuate the proliferation of PC cells, tumor growth, and angiogenesis.

### RNA-seq analysis revealed a differential transcriptome profile in AGTPBP1 knockdown PANC-1 cells

**Fig. 5:**
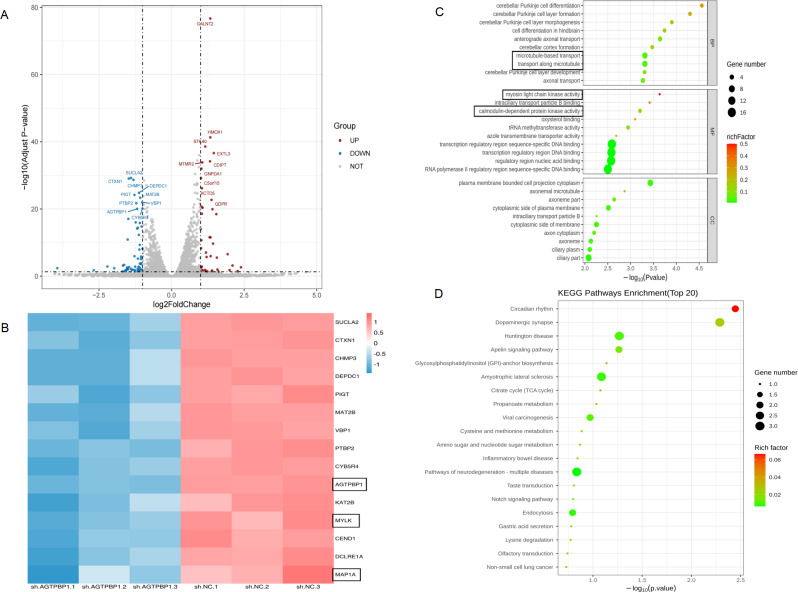
**The differentially expressed genes in the AGTPBP1 KD cells compared to control cells through RNA-seq and bioinformatic analysis.A** A volcano map of differentially expressed genes in the AGTPBP1 knockdown and control groups (*n* = 3/group). Red dots indicate upregulated genes and blue dots indicate downregulated genes. **B** The heat map with significant expression differences between the AGTPBP1 knockdown group and the control group (*n* = 3/group). Red: up-regulated DEGs; blue: down-regulated DEGs. The redder the color, the higher the expression, and the greener, the lower the expression. **C** GO enrichment analysis of significantly differentially expressed genes in the AGTPBP1 knockdown group and the control group cells (*n* = 3/group). **D** Bubble plot of KEGG enrichment analysis of significantly differentially expressed genes in the AGTPBP1 knockdown and control groups (*n* = 3/group). The size of the dot represents the number of genes annotated, and the color from green to red represents the degree of enrichment. $\:P＜0.05$ is considered significantly different.

To determine the mechanism of AGTPBP1 on the regulation of malignant biological behaviors of PC, we performed RNA-seq to compare the gene expression profiles between the AGTPBP1 knockdown PANC-1 cells and negative control cells (*n* = 3). As a result, a total of 292 DEGs were identified, including 186 upregulated and 106 downregulated genes (*P adj* < 0.05,|log2(fold change)|>1). Figure 5A represents the volcano plots of the transcripts. A clustering heat map was then drawn for typical genes with reduced expression levels and significant expression differences (Fig. 5B).

To characterize the biological roles of these DEGs, GO function and KEGG pathway enrichment analysis were used. GO analysis revealed that the DEGs were enriched in the biological process of intracellular microtubule trafficking and the regulation of intracellular myosin light chain kinase activity and calmodulin-dependent protein kinase activity (Fig. 5C). KEGG pathway analysis demonstrated the significantly affected categories in genes that were downregulated or upregulated in response to AGTPBP1 deficiency (Fig. 5D). In addition, we randomly picked 10 DEGs and performed RT-qPCR experiment to confirm their expression (Supplementary Fig. 2). As a result, 8 genes showed consistent expression with RNA-seq results. Among them, the microtubule-associated protein 1 A (MAP1A) and myosin light chain kinase (MYLK) genes were significantly down-regulated in AGTPBP1 KD cells versus control cells (Supplementary Fig. 2).

Taken together, mechanistically, our results suggest that AGTPBP1 is closely related to the PC progression and involved in multiple pathways including intracellular microtubule trafficking, the regulation of intracellular myosin light chain kinase activity, and calmodulin-dependent protein kinase activity.

### AGTPBP1 knockdown inhibits microtubule protein expression and ERK1/2 signaling pathway

**Fig. 6:**
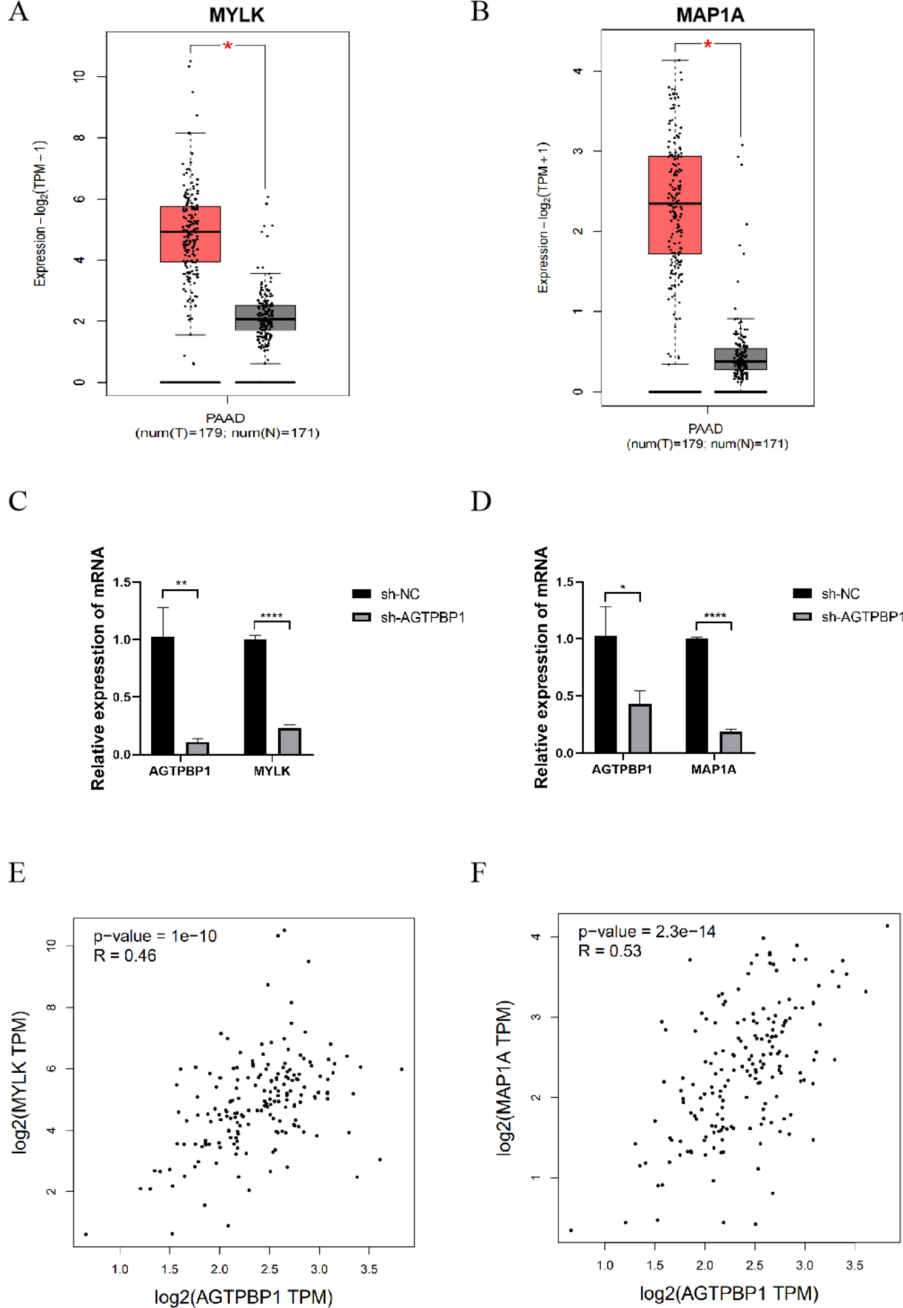
**Expression of MYLK and MAP1A in PC and AGTPBP1 KD cells.A-B** MYLK (**A**) and MAP1A (**B**) were up-regulated in pancreatic cancers based on GEPIA analysis. PAAD, pancreatic ductal adenocarcinoma; T, tumor; N, normal. **C-D** Both MYLK (**C**) and MAP1A (**D**) were down-regulated after AGTPBP1 knockdown in PANC-1 PC cells by RT-qPCR analysis. *n* = 3. **E-F** Positive correlations between the expression of AGTPBP1 and MYLK, AGTPBP1 and MAP1A in PDAC samples were observed by GEPIA analysis. Spearman correlation was used to measure the degree of association between two genes. Values are presented as mean ± SD. The Student’s t-test was used to compare the means between the two groups. *P<0.05, **P<0.01, ****P<0.0001 vs. sh-NC

**Fig. 7:**
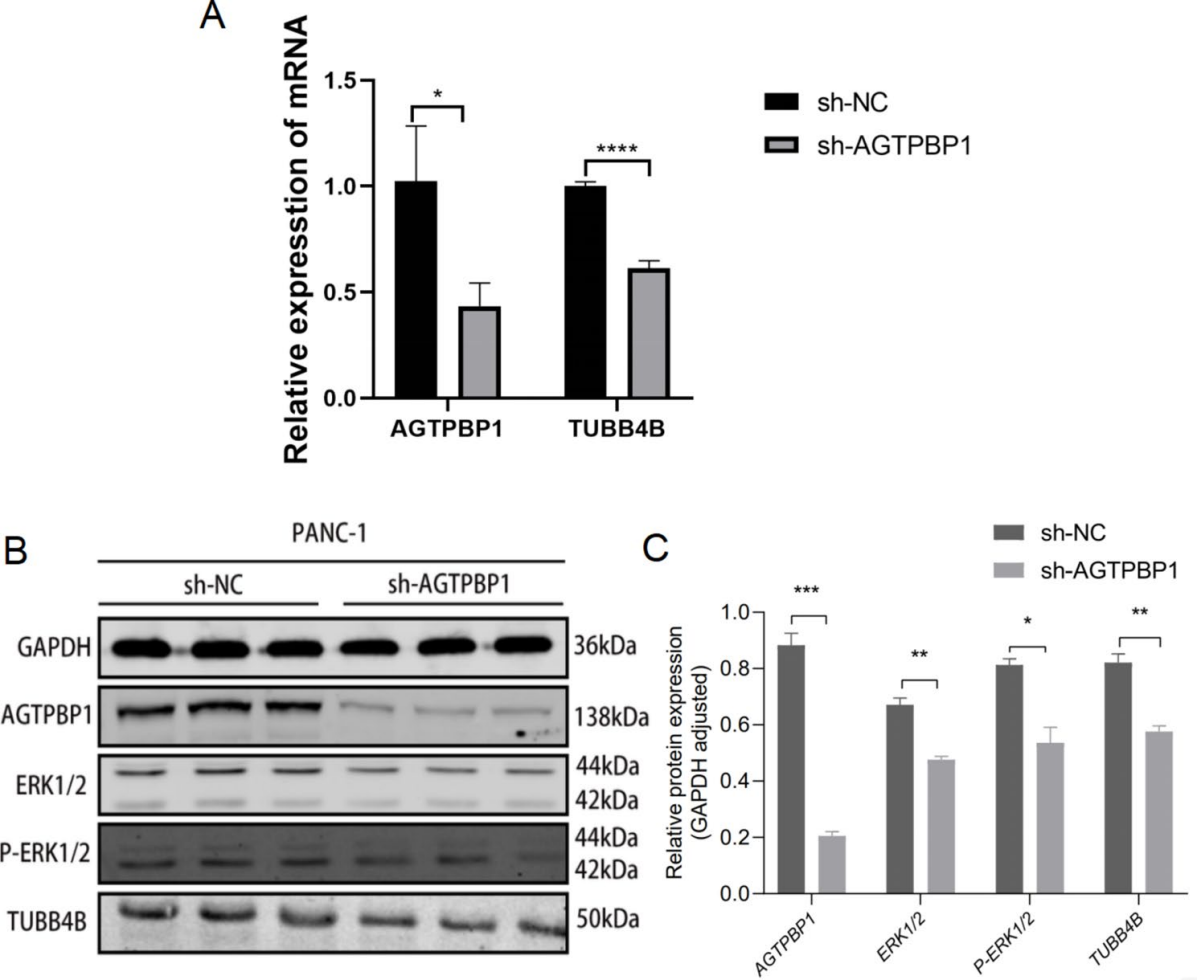
**The expression of TUBB4B**,** ERK1/2**,** and p-ERK1/2 was down-regulated in PANC-1 cells after AGTPBP1 knockdown. A** mRNA level of TUBB4B in AGTPBP1 KD PANC-1 and control cells was detected by RT-qPCR. GAPDH was an internal control. *n* = 3. **B** Protein level of AGTPBP1, TUBB4B, ERK1/2, and p-ERK1/2 in AGTPBP1 KD PANC-1 and control cells was detected by western blot. GAPDH was an internal control. *n* = 3. **C** The gray density of the immunoblot was estimated and normalized with GAPDH to demonstrate the relative expression level of proteins in B. As a result, the knockdown of AGTPBP1 in PANC-1 cells significantly inhibited the expression of TUBB4B, ERK1/2, and p-ERK1/2. Values are presented as mean ± SD. The Student’s t-test was used to compare the means between the two groups. *P<0.05 , **P<0.01 ,*** *P*<0.001, **** *P*<0.0001 vs. sh-NC

According to the previous report, AGTPBP1 possibly regulates MYLK and functions in microtubule stability (Rogowski et al. 2010). The microtubules are crucial in many physiological processes and their dynamics are affected in cancer cells. Thus, based on the literature and our analysis, we would expect to find alterations in microtubule-related proteins such as MAPs and tubulins during cancer progression. Interestingly, we found that MAP1A and MYLK genes were down-regulated in sh-AGTPBP1 cells from RNA-seq and RT-qPCR analysis (Fig. 5B, Supplementary Fig. 2). According to the GEPIA analysis based on TCGA, similar to AGTPBP1, the expression of MYLK and MAP1A in pancreatic cancer tissues was significantly upregulated (Fig. 6A and B) (*P* < 0.05). As we observed, both genes were down-regulated after AGTPBP1 was knockdown in PANC-1 cells (Fig. 6C). The Spearman correlation analysis showed AGTPBP1 was positively correlated with the expression of MYLK and MAP1A in pancreatic cancer tissues by GEPIA analysis (Fig. 6D and E). β-tubulin 4B (TUBB4B), a protein related to microtubules, is highly expressed in PC. Additionally, based on literature predictions, it was suggested to be one of the substrates of AGTPBP1 (Sharbeen et al. 2016; Liu et al. 2023). Therefore, we conducted a preliminary examination of the impact of AGTPBP1 on TUBB4B. We found that the expression of TUBB4B was significantly inhibited after the expression of AGTPBP1 decreased, as confirmed by qRT-PCR (Fig. 7A). Therefore, the correlation of AGTPBP1 and microtubule structural proteins indicated that they may participate in the microtubule stability in PC.

In breast cancer, a reduction in MYLK activity has been linked to activation of the extracellular signal-regulated kinase (ERK1/2) pathway. Phosphorylation of ERK then leads to the phosphorylation of MLCK and myosin light chain (MLC). Additionally, this results in an increased sensitivity to calmodulin, promoting cell migration and contributing to tumor progression (Klemke et al. 1997). Thus, we examined the expression of ERK1/2 and p-ERK1/2 in AGTPBP1 KD cells by western blot analysis. The result showed that the knockdown of AGTPBP1 in PANC-1 cells significantly inhibited the expression of ERK1/2 and p-ERK1/2 (Fig. 7A and B). It indicated that decreased expression of AGTPBP1 attenuated the progression of PC by inhibiting the ERK/MYLK signaling pathway.

## Discussion

PDAC is a clinically challenging cancer, due to its difficulty in early diagnosis, poor surgical outcome, chemotherapy resistance, and metastasis. Deciphering the underlying molecular mechanisms and identifying potential diagnosis and prognosis biomarkers for PDAC will provide new opportunities for early detection and targeted therapy of PDAC. The AGTPBP1 encodes cytosolic carboxypeptidase 1 (CCP1) and functions in the deglutamylation of tubulin. It regulates the assembly, transport, and signaling of microtubules, thereby maintaining the stability and function of the cytoskeletal structure(Rodríguez de la Vega Otazo et al. 2013). Kwak et al. found that a low level of AGTPBP1 expression was associated with poor prognosis in lung cancer patients (Kwak et al. 2020). However, the function of AGTPBP1 in cancers has not yet been fully understood. Our study investigated the expression and roles of AGTPBP1 in pancreatic cancer for the first time.

In this study, we found that AGTPBP1 was overexpressed in PC tissues and cells. Our findings indicated a strong correlation between AGTPBP1 expression and tumor location, particularly with higher expression levels in tumors at the tail of the pancreas. It suggests that AGTPBP1 may play a critical role in the development and progression of tumors in specific regions of the pancreas. This finding can provide a basis for understanding the biological mechanisms underlying PC development in different regions of the pancreas. It may also lead to the identification of region-specific risk factors in PC.

Our study also demonstrated the inhibitory effect of AGTPBP1 on PC cell proliferation, migration, invasion, and metastasis. These findings are essential for the further development of more effective treatment strategies that can target cancer behaviors, potentially leading to better patient outcomes.

Numerous studies indicate that cancer cell division is characterized by changes in microtubule dynamics and is associated with chromosomal instability, aneuploidy, and the development of drug resistance. Microtubule-associated proteins (MAPs) play a crucial role in regulating microtubule dynamics in cancer cells by degrading or inhibiting microtubules (Wattanathamsan and Pongrakhananon 2022). In addition, cell division and migration are dependent on the cytoskeleton, which is maintained by microtubules, actin-containing microfilaments, and mechanochemical molecules. Thus, these previous findings suggest that AGTPBP1 may play a role in cancer by affecting microtubule stability and tubulin deglutamylation.

The correlation between polyglutamylation and tumorigenesis as well as drug resistance in cancer has been highlighted. For instance, polyglutamylated tubulin has been shown to confer resistance to microtubule-destabilizing drugs such as estramustine and nocodazole (Lu et al. 2013). Conversely, high levels of polyglutamylation have been associated with resistance to the microtubule-stabilizing drug paclitaxel in breast cancer(Lu et al. 2013). Polyglutamylation is associated with stable microtubules and several observations suggest that this post-translational modification (PTM) might increase microtubule stability(Westermann and Weber 2003). The study suggests that there is a connection between tubulin glutamylation and cancer. Our analysis indicated that the abnormal expression of AGTPBP1 can regulate the level of tubulin PTM, which impacts the stability of the microtubule structure. Therefore, it is suggested that AGTPBP1 may be a promising target for the treatment of human malignancies by regulating tubulin PTM.

GO analysis based on the RNA-seq experiment showed that AGTPBP1 may be involved in the biological process of intracellular microtubule transport, the regulation of intracellular myosin light chain kinase activity, and calmodulin-dependent protein kinase activity. This is consistent with our previous observations that AGTPBP1 may affect the progression of PC by regulating tubulin deglutamination. Further research is needed to confirm the specific mechanisms involved.

Dysregulation of proteins that comprise the cell cytoskeleton and/or microtubule network has been implicated in chemotherapy drug resistance and aggressive disease in different tumor types (Desai and Mitchison 1997). MAP1A, one of the most abundant microtubule-associated proteins in the mammalian brain, plays a crucial role in maintaining the structural stability of microtubules. Generally, MAPs, which interact with microtubules to regulate their dynamic stages, are involved in various cellular processes. MAP1A deficiency has been linked to neurodegeneration such as ataxia, tremors, and late-onset degeneration of cerebellar Purkinje cells (Liu et al. 2015), but its role in cancers remains unclear. MYLK, which encodes the Ca^2+^/calmodulin (CaM)-dependent myosin light-chain kinase (MLCK), phosphorylates the regulatory light chain to initiate contraction in smooth muscle cells (Khapchaev and Shirinsky 2016). MYLK plays a crucial role in various physiological processes related to myosin activation, such as cell adhesion, migration, division, invasion, and metastasis (Khapchaev and Shirinsky 2016). The study from Rogowski Krzysztof et al. indicated that AGTPBP1 not only targets tubulins but can also remove glutamate from the C-terminus of other substrates, including myosin light chain kinase 1 (MLCK1) (Rogowski et al. 2010). MYLK inhibitors can block the invasion and adhesion of human pancreatic cancer cell lines, suggesting that targeting MYLK may be a promising therapeutic strategy for PC cell invasion and metastasis prevention (Kaneko et al. 2002). Currently, research is focused on MYLK in human malignancies, including digestive system tumors and those outside the digestive tract, not just pancreatic cancer (Lin et al. 2018). Numerous investigations have demonstrated that the ERK-MAPK signaling pathway can directly stimulate cell motility and the migration of cancer cells (Guo et al. 2020). Our findings indicate that AGTPBP1 regulated the expression of ERK1/2 and P-ERK1/2 in PANC-1 cells. Based on these observations, we hypothesize that AGTPBP1 may contribute to the development and progression of PC through the ERK/MYLK signaling pathway and affect microtubule structure. However, the specific mechanism underlying this process remains unclear and requires further investigation.

Accumulating evidence indicates the significant role of microtubule post-translational modifications (PTMs) on cancer behavior(Wattanathamsan and Pongrakhananon 2023). Changes in the PTMs of microtubules have a significant impact on their interaction with MAPs and stability. AGTPBP1 has been shown to regulate tubulin deglutamylation during neuronal development (Rodríguez de la Vega Otazo et al. 2013). There is increasing evidence that tubulin PTMs play a crucial role in tumorigenesis and development, with some studies reporting an association between tubulin isoforms. These β-tubulin isotypes are more highly expressed in PC tissues than in paracancerous tissues (Albahde et al. 2021). Also, they are upregulated in PC cell lines (Albahde et al. 2021). βIV-Tubulin isotype includes two subtypes: tubulin βIVa (TUBB4) and βIVb-tubulin (TUBB4B, also named TUBB2C). Studies have implicated βIVa-tubulin or βIVb-tubulin expression with chemoresistance in prostate, breast, ovarian, and lung cancer. TUBB4B plays a role in regulating vinca alkaloid chemosensitivity in PC cells (Sharbeen et al. 2016). Here, we have discovered that TUBB4B was also regulated by AGTPBP1.

However, challenges remain in fully elucidating the mechanisms by which AGTPBP1 influences these proteins and pathways and in translating these findings into clinical applications. Further research is needed to validate the therapeutic potential of targeting AGTPBP1 in PC treatment.

## Conclusions

Our study demonstrated for the first time that AGTPBP1 is highly expressed in pancreatic cancer tissues and cells and its expression has a close relationship with tumor location. Furthermore, we found that the knockdown of AGTPBP1 can significantly suppress the malignant behaviors of PC cells, suggesting that AGTPBP1 may play an important role in the progression of PC by affecting the ERK/MYLK signaling pathway and microtubule structure.

### Electronic supplementary material

Below is the link to the electronic supplementary material.

Supplementary Material 1: The expression of AGTPBP1 in pancreatic cancer and normal pancreatic tissues is based on publicly available databases. **A** AGTPBP1 was identified in the cBioProptal database and revealed to have approximately 7% amplification mutations in pancreatic cancer. **B** The GEPIA database was utilized to analyze the differential expression of AGTPBP1 in pancreatic cancer tissues and normal pancreatic tissues. The red indicates PC tissues and the black indicates normal pancreatic tissues. **C** Human Protein Atlas online database analyzes the differential expression of AGTPBP1 protein in pancreatic cancer and normal pancreatic tissues, which are mainly expressed in endothelial and ductal cells of pancreatic cancer tissues (**D**)

Supplementary Material 2: The confirmation of expression of 10 DEGs identified by RNA-seq using RT-qPCR method. Except for CDIPT and IGFBPL1, 8 genes showed consistency with the results from RNA-seq. Values are presented as mean ± SD. The Student’s t-test was used to compare the means between the two groups.*, **, ***

## Data Availability

The datasets analyzed during the current study are available in the TCGA and the GTEx projects. The data that support the findings of this study are available from the corresponding author upon reasonable request.

## Abbreviations

AGTPBP1: ATP/GTP binding protein 1
PDAC: Pancreatic ductal adenocarcinoma
PC: Pancreatic cancer
IHC: Immunohistochemistry
RT-qPCR: Reverse Transcription quantitative PCR
MYLK: Myosin light chain kinase
MAP1A: Microtubule-associated protein 1 A
TUBB4B: Tubulin, beta 4B class IVb
CCP1: Cytosolic carboxypeptidase 1
CCPs: Cytosolic carboxypeptidases
PTMs: Post-translational modifications
AOD: Average optical density
MAPK: Mitogen-activated protein kinase
ERK: Extracellular regulated protein kinase
