# Supplementary figures and images for: Targeting AGTPBP1 inhibits pancreatic cancer progression via regulating microtubules and ERK signaling pathway
Source: Mol Med. 2024 Aug 11;30:119. doi: 10.1186/s10020-024-00892-x (PMC11318240; doi:10.1186/s10020-024-00892-x)

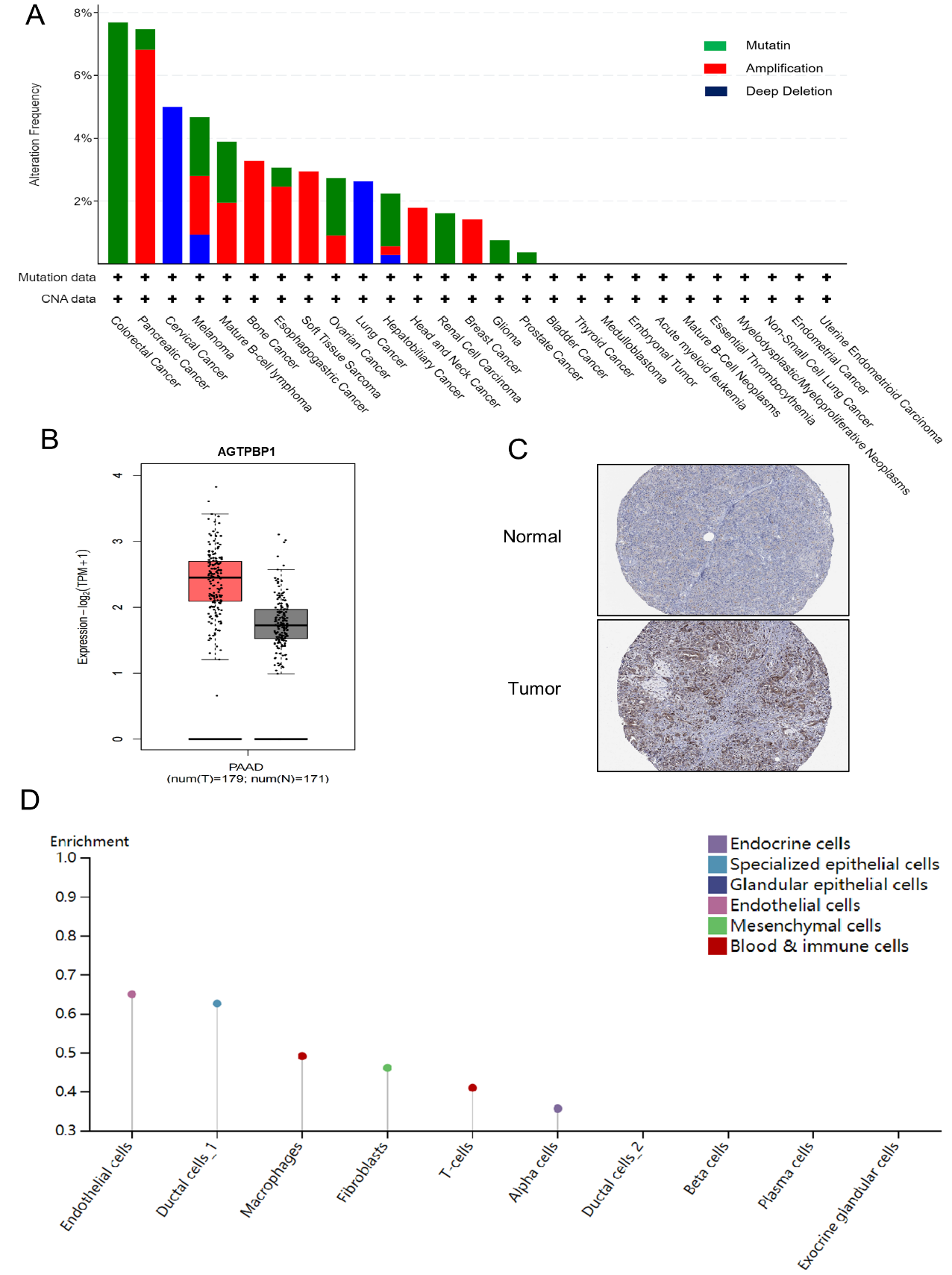

Supplement: Supplementary file 1 — Supplementary Material 1: The expression of AGTPBP1 in pancreatic cancer and normal pancreatic tissues is based on publicly available databases. A AGTPBP1 was identified in the cBioProptal database and revealed to have approximately 7% amplification mutations in pancreatic cancer. B The GEPIA database was utilized to analyze the differential expression of AGTPBP1 in pancreatic cancer tissues and normal pancreatic tissues. The red indicates PC tissues and the black indicates normal pancreatic tissues. C Human Protein Atlas online database analyzes the differential expression of AGTPBP1 protein in pancreatic cancer and normal pancreatic tissues, which are mainly expressed in endothelial and ductal cells of pancreatic cancer tissues (D) [file 10020_2024_892_MOESM1_ESM.tif]

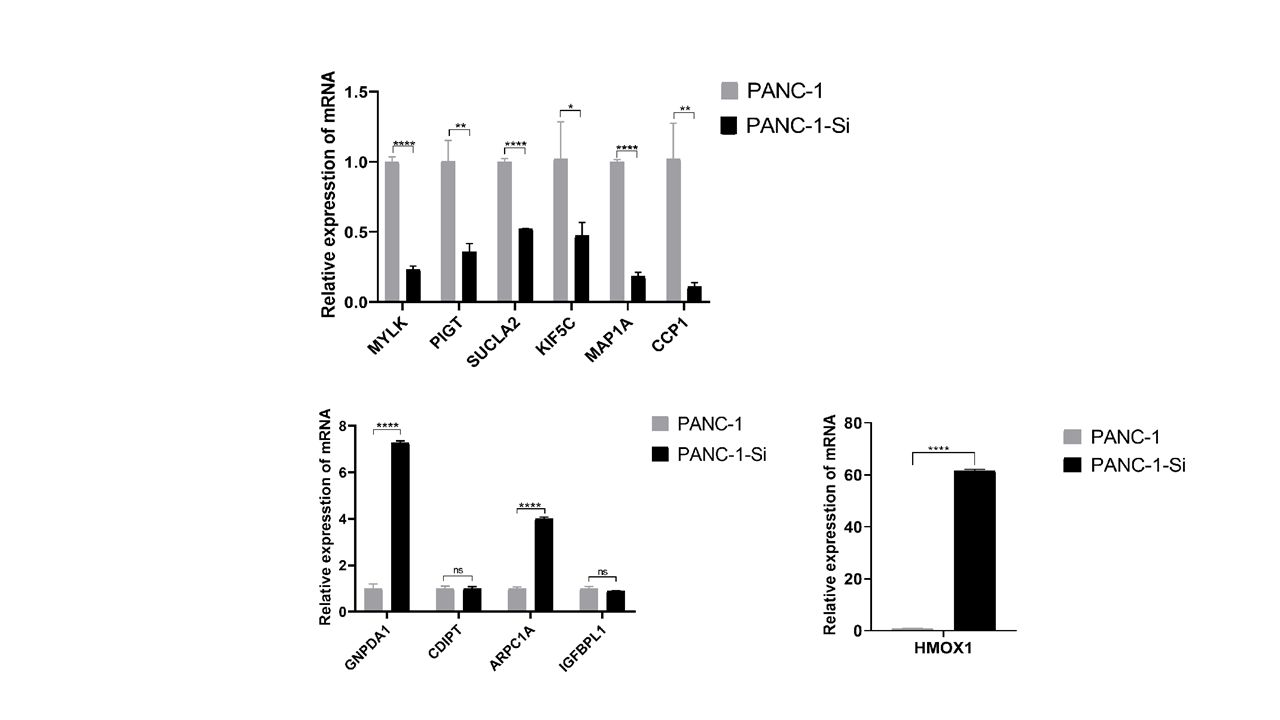

Supplement: Supplementary file 2 — Supplementary Material 2: The confirmation of expression of 10 DEGs identified by RNA-seq using RT-qPCR method. Except for CDIPT and IGFBPL1, 8 genes showed consistency with the results from RNA-seq. Values are presented as mean ± SD. The Student’s t-test was used to compare the means between the two groups.*, **, *** [file 10020_2024_892_MOESM2_ESM.tif]
